# Comprehensive Analysis of Chromatin Accessibility and Transcriptional Landscape Identified BRCA1 Repression as a Potential Pathological Factor for Keloid

**DOI:** 10.3390/polym14163391

**Published:** 2022-08-19

**Authors:** Kuixia Xie, Jingrun Yang, Qianqian Yao, Yang Xu, Yonglin Peng, Xinhua Liu

**Affiliations:** 1 Department of Dermatology, The Affiliated Hospital of Hangzhou Normal University, Hangzhou 310015, China; 2 Department of General Surgery, The First Medical Center of Chinese PLA General Hospital, Beijing 100853, China; 3 Department of Biochemistry and Molecular Biology, School of Basic Medical Sciences, Hangzhou Normal University, Hangzhou 311121, China; 4 Shanghai Center for Systems Biomedicine, Shanghai Jiao Tong University, Shanghai 200240, China

**Keywords:** keloid, ATAC-seq, chromatin accessibility, BRCA1, NPTX2

## Abstract

Keloid is a poorly understood fibrotic skin disease that commonly occurs during wound-healing. As a polymer composed of nucleic acid and proteins, the structure of chromatin could be dynamically regulated in the nucleus. In this study, we explored the dynamics of chromatin accessibility and the transcriptome in dermal fibroblasts (DFs) in keloid formation. Compared to normal samples, chromatin accessibility and transcriptome were extensively altered in keloid DFs. In addition, changes in chromatin accessibility were closely associated with changes in gene expression in DFs. Breast cancer type 1 (BRCA1) was significantly downregulated in keloid DFs, and its knockdown promoted the proliferation and attenuated the migration ability of normal DF cells. Mechanistically, BRCA1 suppression significantly reduced the expression of neuronal pentraxin 2 (NPTX2), a cell viability-related gene. BRCA1 binding affinity at the NPTX2 enhancer and the chromatin accessibility in the same region were significantly lower in keloid DFs than in normal DFs, which might contribute to NPTX2 inhibition. In conclusion, this study identified BRCA1 inhibition in DFs as a novel pathological factor in keloids and preliminarily explored its potential mechanisms, which will help us understand the formation of keloids.

## 1. Introduction

Wound-healing is a complex process which could be typically divided into three successive overlapping phases: inflammation, proliferation, and remodeling [1]. In the wound microenvironment, strict and frequent interactions, which are fundamental for cell behavior decisions occur among different cell types, such as keratinocytes, fibroblasts, and immune cells [2,3]. For example, the wound undergoes the dense recruitment of immune cells during the inflammatory phase, and cytokines secreted by these immune cells activate fibroblasts to produce the extracellular matrix (ECM) and collagen [4]. Keratinocytes comply with dermal fibroblast (DF) signals, migrating and proliferating in a paracrine manner during the remodeling phase [5]. Aberrant cell interactions can contribute to abnormal wound-healing, resulting in defective or excessive healing. Keloid is a common fibrotic skin disease caused by excessive ECM and collagen deposition, which is characterized by scars beyond the original wound margin. Keloids can disfigure or even disable the pathogenic part, and it is prone to relapse after treatment. Currently, there is still a lack of effective therapeutic methods [6]. Some molecular events, such as the activation of the transforming growth factor-beta (TGF-β) signaling pathway, are involved in keloids formation [7]. However, our understanding of the underlying mechanisms of keloids remains limited, which is an obstacle to the development of effective therapies.

The morbidity of keloid differs among ethnicities. The prevalence of keloid in populations with darker skin is significantly higher than that in demographics with lighter skin and is heredofamilial, implying the importance of genetics in keloids [8]. Several genome-wide linkage studies have identified some genomic loci whose variations were statistically associated with keloid risk, such as the 2q23 and 7p11 chromosome regions in Japanese and African American families [9] and 15q22.31–q23, 18q21.1, and 10q23.31 in Chinese families [10]. However, the high genetic heterogeneity among different patients makes it difficult to develop target loci. Epigenetic regulation is crucial in determining gene expression through various mechanisms without gene sequence alterations, such as non-coding RNA regulation and chromatin modification. Perturbed epigenetic regulation can induce aberrant gene expression and underlies multiple diseases. Various epigenetic perturbations have been implicated in keloid formation, including long or small non-coding RNA dysregulation, abnormal DNA methylation, and histone modifications [11]. Chromatin is a polymer with different hierarchical structures. Chromatin accessibility represents the most basic structure and the foundation of gene expression regulation by influencing the binding of transcription factors (TFs) to the cis-regulating elements of genes, which is regulated by multiple epigenetic mechanisms. For example, the histone acetylation and binding of pioneer TFs can induce an open chromatin state, namely, more accessible chromatin for TFs, and is closely associated with transcriptional activation [12,13,14]. Alterations in chromatin accessibility have been extensively reported in the pathogenesis of multiple diseases, including cancer and autoimmune and inflammatory disorders [15,16,17]. However, the exploration and understanding of chromatin state transformation in keloid formation is currently limited.

In this study, we comprehensively analyzed and compared chromatin state alterations in the DFs of keloid and normal samples by combining an assay of transposase accessible chromatin sequencing (ATAC-seq) and a series of histone modification ChIP-seq data. We identified breast cancer type 1 (BRCA1) as a potential keloid-associated gene by integrating gene expression and cell experiments.

## 2. Materials and Methods

### 2.1. Study Subject

The derma of keloids and adjacent normal tissues on the back were obtained from a 67-year-old man with signed informed consent. The ethics committee of the PLA General Hospital approved this study (Ethics Approval NO.: S2018–223–02).

### 2.2. DF Extraction and Cell Culture

DFs were isolated from keloid tissues and paired normal skin using an explant technique [18]. Briefly, dermal tissues were washed three times with phosphate-buffered saline (PBS) (CellMax Inc, Sunnyvale, Sweden) and minced into small pieces (~1 mm). They were explanted in Dulbecco’s modified Eagle medium (DMEM) (CellMax Inc, Sunnyvale, Sweden) containing 10% fetal bovine serum (FBS) (CellMax Inc, Sunnyvale, Sweden) and 1% antibiotic–antimycotic solution (Gibco, Invitrogen Gibco Inc, Waltham, MA, USA) and incubated at 37 °C in 5% carbon dioxide. The medium was changed every two days. After 7–10 days, cells proliferating from the edge of the tissues were regarded as cultured fibroblasts. The cells were passaged 1–2 times per week.

### 2.3. BRCA1 Knockdown

Recombinant lentiviruses expressing BRCA1 short hairpin RNA (shBRCA1) were constructed by Gene Pharma (Shanghai, China). Concentrated viruses were used to infect 5 × 10^5^ cells in 6-well plates with 6 μg/mL polybrene. After two days, puromycin was added to the medium at a concentration of 2 μg/mL to select stably-transduced cells. The shBRCA1 sequence was GAGTATGCAAACAGCTATAAT.

### 2.4. Reverse Transcription-Polymerase Chain Reaction (RT-PCR)

Total RNA was extracted using the SteadyPure Quick RNA Extraction Kit (AG) according to the manufacturer’s instructions and used for first-strand cDNA synthesis using the Evo M-MLV RT Mix Kit (AG). The quantification of all gene transcripts was performed on a QuantStudio™5 Real-Time PCR Instrument (Applied Biosystems Inc, Foster City, CA, USA) using the SYBR^®^ Green Premix Pro Taq HS qPCR Kit (Rox Plus, Hu’nan, China) (AG), and RNA levels were normalized to those of 18S rRNA. 2^−^^ΔΔ^^ct^ was applied to analyze the data. Three parallel duplicate wells were designed for the experiment, and all samples were tested three times. Error bars represent the mean ± standard deviation (SD) from three independent experiments. The primer sequences used were as follows: 18S rRNA, forward: 5′-GTAACCCGTTGAACCCCATT-3′, reverse: 5′-CCATCCAATCGGTAGTAGCG-3′; BRCA1, forward: 5′-AGGAACCTGTCTCCACAAAGTG-3′, reverse: 5′-TCGTACTTTCTTGTAGGCTCCTTT-3′; and NPTX2, forward: 5′-AGAAGTCCCTGCTGCACAAT-3′, reverse: 5′-TTAAAGGCGCTATTGCCTCGC-3′.

### 2.5. ATAC-Seq

ATAC-seq was performed using the Chromatin Profile Kit for Illumina N248 (Novoprotein Inc., Tianjin, China), according to the manufacturer’s instructions. The libraries were subjected to paired-end 150 bp sequencing on a NovaSeq platform at GENEWIZ (Suzhou, China).

### 2.6. Cleavage under Targets and Tagmentation Sequencing (CUT&Tag-Seq)

A CUT&Tag assay was performed using the NovoNGS^®^ CUT&Tag 3.0 High-Sensitivity Kit N259-YH01 (NovoProtein Inc., Tianjin, China) according to the manufacturer’s instructions. Libraries were sequenced by GENEWIZ (Suzhou, China) using paired-end 150 bp sequencing on the NovaSeq platform. The following antibodies were used: BRCA1 (sc-6954) and normal mouse immunoglobulin G (IgG) (sc-2025) from Santa Cruz Biotechnology; H3K4me3 (ab8580), H3K4me1 (ab8895), and H3K9me3 (ab8898) from Abcam; H3K27ac (#8173) and normal rabbit IgG (#2729) from Cell Signaling Technology; donkey anti-mouse IgG-AlexaFluor 288 (abs20014) from Abcam; and DyLight 488 AffiniPure Goat Anti-Rabbit IgG (H+L) (E032220) from EarthOx.

### 2.7. RNA-Seq

RNA libraries were constructed using total RNA and were sequenced using paired-end 150 bp sequencing on the NovoSeq platform at GENEWIZ (Suzhou, China).

### 2.8. ATAC-Seq Analysis

Raw data were preprocessed using fastp version 0.19.6 [19] for adapters, low-quality bases, and read removal. The preprocessed data were aligned to the GRCh38/hg38 reference genome using Bowtie version 2.4.3 [20] with a tolerance of one mismatch at most and a 2000 bp maximum fragment length. Picard version 2.26.11 (https://broadinstitute.github.io/picard/, accessed on 22 November 2021) was applied to remove duplicated mappings and samtools version 1.15 removed the multiple alignments (https://www.htslib.org/doc/samtools.html, accessed on 22 November 2021). The model-based analysis of the ChIP-seq version 2.2.7.1 [21] was used to identify nucleosome-free chromatin regions based on a *q*-value < 0.05 threshold. Chromatin regions were annotated using the ChIPseeker R package [22] with a promoter defined as a 3000 bp chromatin interval centered on the transcriptional start site (TSS). Differentially accessible analysis was conducted using MAnorm [23] with the thresholds of an absolute log2-based signal ratio >1 and a *p*-value < 0.05. The functional enrichment analysis of differentially accessible regions was based on the Genomic Regions Enrichment of Annotations Tool (GREAT) version 4.0.4 [24] with a *p*-value < 0.05.

### 2.9. CUT&Tag-Seq Analysis

The preprocessing and alignment methods for CUT&Tag-seq data were the same as those used for ATAC-seq. However, the duplicated mappings were retained because they are not PCR-biased, as in the traditional ChIP-seq library construction protocol, but have biological meanings. The peak calling of both BRCA1 and histone modifications was conducted by using Sparse Enrichment Analysis for CUT&RUN (23 November 2021, SEACR) [25] with IgG as a control for read density normalization. The “stringent” mode was used for peak significance determination. The ChIPseeker R package was used for peak annotation with ATAC-seq. The DeepTools toolkit [26] was used to calculate, normalize, and visualize the CUT&Tag-seq and ATAC-seq signals across the specified chromatin regions.

### 2.10. RNA-Seq Analysis

Fastp version 0.19.6, used for ATAC-seq data, was used to preprocess the raw sequencing data. Spliced Transcripts Alignment to a Reference (STAR) version 2.7.10a [27] was used to align the preprocessed data to the GRCh38/hg38 reference genome with the default parameters. HTSeq version 2.0.1 [28] was used to calculate the read number mapped to the exons of each gene. Differential gene expression analysis was performed using the DESeq2 R package [29] with a threshold of *p* < 0.05 and absolute log2-based fold change >1. The functional enrichment analysis of differentially expressed genes (DEGs) was conducted based on the Database for Annotation, Visualization, and Integrated Discovery (DAVID) 2021 update (https://david.ncifcrf.gov/, accessed on 30 November 2021) with a *p*-value < 0.05.

## 3. Results

### 3.1. Keloid DFs Exhibited Extensive Chromatin State Changes

**Figure 1:**
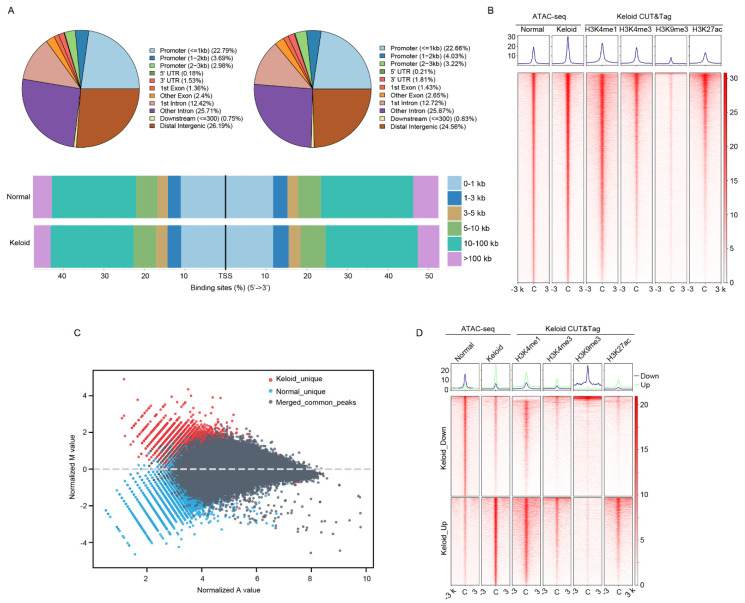
Comparative analysis of chromatin accessibility landscape between dermal fibroblast (DF) of keloid and normal skin tissues. (**A**) Genomic feature distribution of accessible, namely nucleosome-free, chromatin regions in normal (upper left panel) and keloid (upper right panel) DF. The bottom panel provides a stack plot illustrating the proportions of different sets of accessible chromatin regions stratified by their distance to the gene’s transcriptional start site (TSS). (**B**) Signal profiles of ATAC-seq in normal and keloid DF and CUT&Tag-seq of a series of histone modifications in keloid DF across the accessible chromatin regions that annotated as “distal intergenic” in normal and keloid DF. (**C**) MA-plot illustrating the normalized mean ATAC-seq signal values of keloids and normal DF (A values in X-axis) and log2-based ratio of ATAC-seq signal values of keloid DF to normal DF (M values in Y-axis) across the combined accessible chromatin regions in normal and keloid DF samples. Red and blue dots represent chromatin regions that are accessible only in keloids and normal DF, respectively. Dark grey dots are the merged chromatin regions of overlapped accessible regions in normal and keloid DF. (**D**) Signal profiles of ATAC-seq in normal and keloid DF and CUT&Tag-seq of a series of histone modifications in keloid DF across the differentially accessible regions stratified by their accessible state in keloid DF relative to normal DF. Keloid_down and keloid_up represents chromatin regions that are significantly tighter and looser in keloids than normal samples, respectively.

A high-throughput ATAC-seq assay was applied to the DF samples from keloid and adjacent normal tissues to acquire their accessible chromatin regions, namely those without nucleosome encapsulation. Quality control in terms of sequencing fragment length distribution indicated that the high-quality sequencing library for the nucleosome-free and mono-, di-, and tri-nucleosome fragments predominated the library pool (Figure S1). Peak calling statistically identified 100,952 and 92,208 accessible chromatin regions in keloid and normal DFs, respectively. As Figure 1A shows, promoter, intron, and distal intergenic regions represent the most enriched genomic features of the accessible regions. We performed high-throughput CUT&Tag-seq for various histone modifications, including H3K4me1, H3K4me3, H3K9me3, and H3K27ac, in keloid DFs and calculated their signals at “distal intergenic”-annotated chromatin regions to determine whether those distal intergenic regions were enhancers. H3K4me1, a robust enhancer marker, was prominently enriched in the distal intergenic regions along with H3K27ac, an active chromatin marker (Figure 1B). The heterochromatin marker H3K9me3 was significantly depleted in distal intergenic regions (Figure 1B). This indicated that the accessible distal intergenic regions were active enhancers. We conducted a differentially accessible region analysis to estimate the chromatin state changes in keloid formation, which statistically identified a total of 4951 tighter and 4347 looser chromatin regions in DFs from keloids compared with those from normal tissues (Figure 1C). The stratification analysis of histone modification signals showed significant H3K27ac enrichment and depletion in keloid samples at chromatin regions that were looser and tighter, respectively. This demonstrated the reliability of the differentially accessible analysis (Figure 1D). Additionally, the H3K4me1 signal showed strong enrichment across these differential chromatin regions but was biased to the looser regions in keloids, implying that enhancers made up most of the perturbed chromatin regions in keloid formation (Figure 1D). Functional interpretation of the looser (Figure S2A) and tighter regions (Figure S2B) identified many keloid-related pathways, such as epithelial cell migration and collagen metabolic processes, implicating the importance of chromatin accessibility changes in keloid formation.

### 3.2. Chromatin State Transformation Is Related to Gene Expression Changes in Keloid Formation

**Figure 2:**
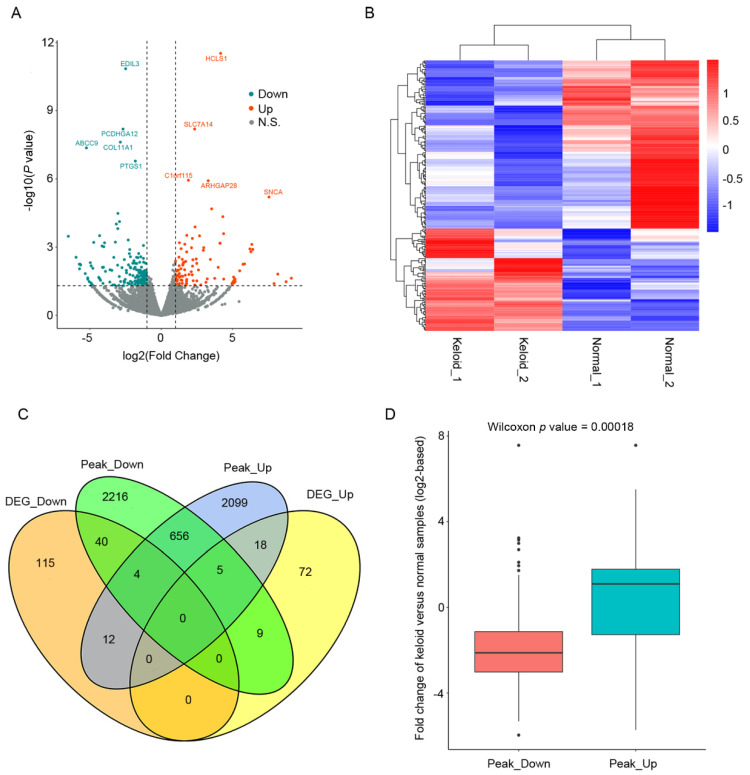
Integrated analysis of chromatin accessibility and transcriptional landscape uncovered influence of chromatin state transformation on gene expression alteration in keloid formation. (**A**) Volcano plot showing the log2-based fold change (X-axis) and significance (Y-axis, −log10 (*p* value)) of differential gene expression analysis between keloids and normal DF samples. Blue and red dots represent genes that are significantly down- and upregulated in keloid DF relative to normal DF, respectively. Dark grey dots are non-significantly differential expression genes. (**B**) Heatmap illustrating the z-score normalized expression values of the differentially expressed genes (DEGs) in normal and keloid DF samples. (**C**) Overlaps between DEGs and differentially accessible chromatin region-associated genes stratified by their changes in keloid DF relative to normal DF. Peak_Down and Peak_Up represent genes annotated by chromatin regions that are tighter and looser in keloid DF compared with normal DF, respectively. DEG_Down and DEG_Up are genes that are significantly down- and upregulated in keloid DF compared with normal DF, respectively. (**D**) Distribution of log2-based fold changes of Peak_Down and Peak_Up genes in keloid DF relative to normal DF.

RNA-seq was performed on DF samples from keloids and normal tissues. We identified 171 significantly downregulated and 104 upregulated genes in keloid samples. Figure 2A shows a volcano plot of the differential expression analysis with the ten most significant genes labeled. Previous reports have implicated some of the DEGs in keloids or other fibrotic diseases, such as collagen genes in keloids [30] and SNCA in kidney fibrosis [31]. Figure 2B shows the relative gene expression levels of these DEGs as a heatmap. The functional enrichment analysis of these DEGs identified many keloid-related pathways (Figure S3A) and biological processes (Figure S3B), including focal adhesion, ECM-receptor interaction, and apoptotic process regulation. We performed a cross-analysis between gene expression and chromatin accessibility to explore whether gene expression changes were associated with chromatin state transformation in keloid formation. Looser and tighter regions in the keloid DF samples were annotated to their nearest genes, followed by intersection analysis with DEGs. There were 23 and 14 intersections of upregulated genes with looser and tighter regions and 44 and 16 intersections of downregulated genes with tighter and looser regions, respectively (Figure 2C). Additionally, the expression fold changes of looser and tighter region-associated genes were significantly different. Briefly, genes within looser and tighter regions tended to be up- and downregulated in keloid samples, respectively (Figure 2D). A more accessible chromatin state was associated with more active gene expression and vice versa. These results indicate that the chromatin state transformation could, at least partially, underlie gene expression changes in keloid formation.

### 3.3. BRCA1 Suppression Is Potentially Associated with Keloid Pathogenesis

**Figure 3:**
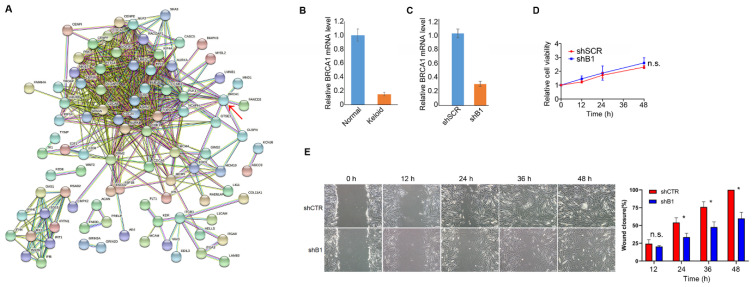
Suppressed BRCA1 expression is a potential pathogenic factor in keloid. (**A**) Protein–protein interaction (PPI) network of DEGs based on STRING database. More lines between two genes indicates more reliable interactions between them, and line color represents evidence type for their interaction. BRCA1 as a hub gene, i.e., highly connected in the network, is highlighted by a red arrow. (**B**) Validation of down-regulation of BRCA1 in keloid DF compared with normal DF, as well as in shBRCA1 and shSCR DF samples by RT-PCR. (**C**) Validation of the suppression of BRCA1 by small hairpin RNA (shRNA) by RT-PCR in normal DF. (**D**) Relative cell viability of normal DF that transfected with shRNA for BRCA1 (shB1) or control (shSCR) quantified by CCK-8 method. (**E**) Wound-healing ability of normal DF that transfected with shB1 or shSCR and quantified by scratch test. The left panel shows the representative DF wound-healing examples and the right panel illustrating the wound closure percent of shB1 and shSCR DF at different time points. Error bars represent the mean ± standard deviation (SD) of three independent experiments. n.s., not significant; * *p* value < 0.05.

We performed a protein–protein interaction (PPI) analysis of 275 DEGs, which divided the DEGs into two densely connected gene groups (Figure 3A). BRCA1, a well-known tumor suppressor with high mutation rates in multiple cancers that was downregulated in keloid samples in this study, is highly connected to the PPI network. We confirmed its downregulation in keloid samples by RT-PCR (Figure 3B). To investigate whether its suppression was associated with DF behavior, we transfected normal DFs with shBRCA1 or control short hairpin RNA (shSCR) and performed cell viability and wound-healing assays. ShBRCA1 significantly repressed BRCA1 expression (Figure 3C). As a result, the cell viability assay indicated no significant chance of the proliferative ability of DFs after BRCA1 inhibition (Figure 3D). However, BRCA1 inhibition significantly attenuated the migration ability (Figure 3E) of normal DFs. Effective DF migration is a prerequisite for efficient wound-healing. We inferred that BRCA1 suppression might induce keloids by promoting DF deposition and prolonging the wound-healing process.

**Figure 4:**
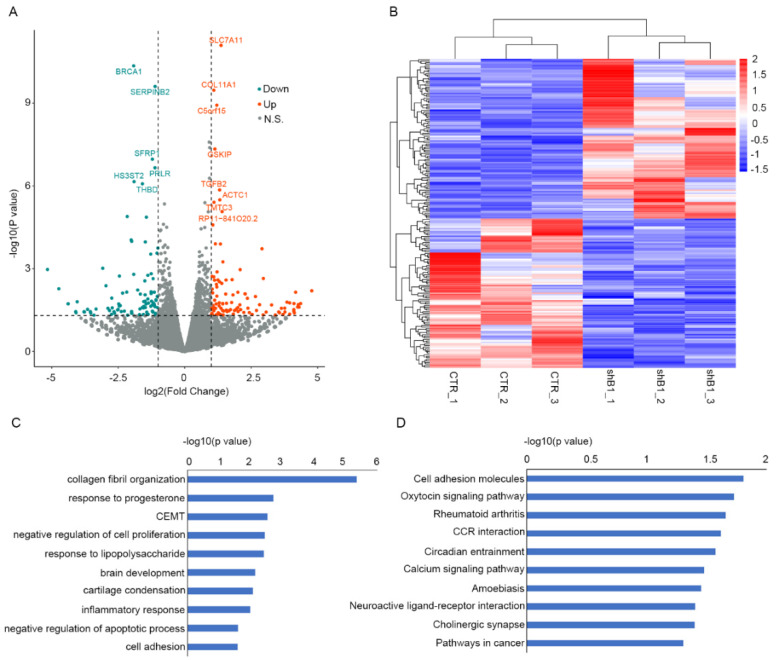
BRCA1 associates with molecular events related to keloid formation in normal DF. (**A**) Volcano plot illustrating the log2-based fold change (X-axis) and significance (Y-axis, log10-based *p* value) of differential expression analysis between shB1 and shSCR DF samples. Blue and red dots are significantly down- and upregulated genes in normal shB1 DF relative to normal shSCR DF, respectively. Dark grey dots represent non-significantly differential expression genes. (**B**) Heatmap of z-score normalized expression values of DEGs in normal shB1 and shSCR DF. (**C**) and (**D**) represent the significantly enriched GO terms and KEGG pathways of DEGs, respectively. CEMT, cardiac epithelial to mesenchymal transition.

We conducted an RNA-seq of normal shBRCA1 and shSCR DFs to explore the molecular influences of BRCA1 inhibition on normal DFs, which revealed 97 downregulated and 104 upregulated genes in shBRCA1 DF samples (Figure 4A,B). The functional enrichment analysis of these DEGs revealed many Gene Ontology (GO) terms and pathways (Figure 4C,D) that could influence keloid formation, such as collagen fibril organization and the negative regulation of cell proliferation. Additionally, Gene Set Enrichment Analysis (GSEA) identified many activated and repressed hallmarks associated with keloid formation after BRCA1 inhibition (Figure S4). This implies that BRCA1 suppression could induce the perturbation of keloid-related molecular events in normal DFs, potentially representing a keloid pathogenesis factor.

### 3.4. NPTX2 Is a Potential Target in Keloid Formation Induced by BRCA1 Suppression

**Figure 5:**
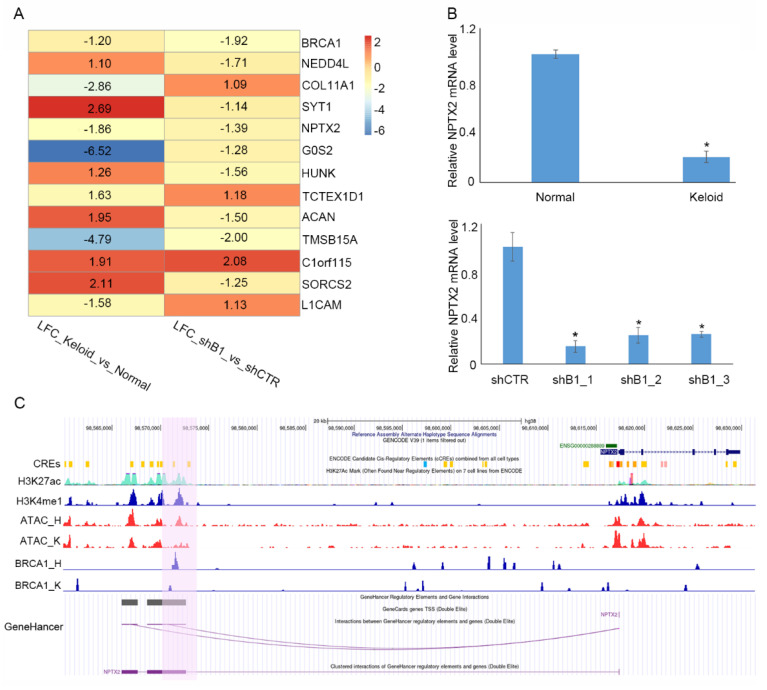
BRCA1 might regulate NPTX2 expression by manipulating its enhancer accessibility and influencing enhancer–promoter interactions in normal DF. (**A**) Heatmap of the log2-based fold change of genes that significantly affected differential expression in both shB1 normal DF compared with shSCR normal DF and keloid DF compared with normal DF. LFC, log2-based fold change. (**B**) Validation of down-regulation of NPTX2 in keloid DF relative to normal DF (upper panel) and shB1 normal DF relative to shSCR normal DF (bottom panel). (**C**) Density profile of H3K27ac and H3K4me1 CUT&Tag-seq in keloid DF, ATAC-seq in normal and keloid DF, BRCA1 CUT&Tag-seq in normal and keloid DF at NPTX2 as well as its associated enhancer region. Interaction between NPTX2 enhancer and promoter predicted based on GeneHancer database is shown in bottom. H, normal DF; K, keloid DF. The promoter-associated enhancer that exhibits looser chromatin organization and higher BRCA1 binding signal is highlighted by purple background. ShB1, shBRCA1.

Previous reports have implicated BRCA1 in transcriptional regulation through multiple molecular mechanisms. Therefore, we explored whether BRCA1 suppression induced keloid formation by regulating other genes. We intersected DEGs in keloid samples and shBRCA1 normal DF samples and obtained 13 overlaps, including some well-known keloid or other fibrotic disease-related genes, such as COL11A1 [32] and NEDD4L [33] (Figure 5A). Interestingly, NPTX2, a synapse formation-related gene recently implicated in abnormal cell proliferation and migration in multiple cancers [34,35], was significantly inhibited in keloids and shBRCA1 normal DF samples (Figure 5B). This indicated that BRCA1 might influence DF behavior by regulating NPTX2. We performed a BRCA1 CUT&Tag-seq in keloids and normal DFs to further investigate the regulatory relationship between BRCA1 and NPTX2. We found a significant BRCA1 binding signal in the NPTX2 enhancer region of the normal DFs but not keloid DFs (Figure 5C). Furthermore, the ATAC-seq signal in this region in normal DFs was higher than that in keloid DFs. The GeneHancer database [36] illustrated a reliable interaction between this enhancer and the NPTX2 promoter (Figure 5C). Given the previous reports on BRCA1 in chromatin remodeling [37], we proposed that BRCA1 suppression in keloids attenuated the accessibility of the NPTX2 enhancer and dismissed the interaction between the NPTX2 enhancer and promoter, resulting in NPTX2 repression. To further validate the role of BRCA1 in chromatin remodeling, we visualized the binding signal of BRCA1 at TP63, a popular target of BRCA1, in both the normal and keloid DF samples along with the H3K4me1 and H3K27ac CUT&Tag as well as the ATAC-seq signals. As a result, consistent with the signal landscape at NPTX2, the binding signal of BRCA1 at TP63 is lower in keloid DF than normal DF and the chromatin is denser at the corresponding region in keloid DF (Figure S5).

## 4. Discussion

Keloid is a common fibrotic skin disease, but its underlying mechanisms are poorly understood. Here, we report that alterations in chromatin accessibility may underlie keloid formation, and BRCA1 suppression might contribute to the regulation of keloid formation by manipulating the accessibility of the amplifier by NPTX2.

Abnormal cellular behaviors, such as the excessive proliferation of fibroblast induced by impaired communication among keratinocytes, immune cells, and DFs, represent the most popular interpretation of keloid formation in wound-healing [5,38]. However, the underlying molecular mechanisms are not fully understood. An open chromatin environment, namely a nucleosome-free environment, is a prerequisite for normal gene expression because most TFs cannot bind directly to nucleosomes. Aberrant chromatin-accessible states, containing looser and tighter nucleosome organization, constitute many pathological cellular behaviors and could result in homeostasis loss, giving rise to multiple disorder states, including cancer and fibrotic diseases [39,40]. Many factors are implicated in the regulation of chromatin accessibility, including the linkage of pioneering factor, histone modification, DNA methylation, and sequential mutations [41,42,43], some of which are thought to be involved in the formation of keloids [44]. However, comprehensive studies on alterations in chromatin accessibility in keloids have rarely been reported. In this study, we compared the state of chromatin on a genome-wide basis between keloid and normal DFs using ATAC-seq and identified numerous chromatin regions that were more or less well organized in keloid samples. By combining the signals of various histone modifications quantified by CUT&Tag-seq, we found that most of these differential chromatin regions were cis-regulating elements, including H3K4me1-enriched enhancers and H3K4me3 enriched promoters, which laid the foundation for transcriptional gene regulation. Additionally, as expected, changes in the level of chromatin accessibility were positively correlated to changes in gene expression in keloids, implying that alterations in chromatin state could at least partially underlie the changes in gene expression in keloid formation. Therefore, the identification of chromatin state regulators in the formation of keloids could shed new light on its pathogenesis and help develop therapeutic targets.

One of the most studied tumor suppressors, BRCA1, has mutations that are closely related to the incidence rate of breast cancer and ovarian cancer [45]. Its most well-known molecular function is DNA damage repair, which is essential for maintaining genome stability [46]. Recent investigations have also uncovered its role in regulating chromatin organization and gene expression [47]. In this study, BRCA1 expression was significantly repressed in keloid samples, implying that BRCA1 might inhibit keloid formation in the wound-healing process. This conclusion is also supported by the enhanced proliferation capacity of normal DFs induced by BRCA1 inhibition. Unexpectedly, BRCA1 inhibition significantly repressed the migration ability of normal DFs, meaning that BRCA1 should promote DF migration during wound-healing, a cell event that might enhance keloid formation, according to some previous studies [48,49]. Wound-healing is a complex process involving inflammation, proliferation, and remodeling. DFs are attracted to the wound site during the inflammatory phase by platelet-derived growth factors (PDGFs) and interleukin (IL)-1β and secrete collagen and other ECMs necessary for wound closure [50]. Additionally, DFs recruited on the wound bed produce growth factors that can stimulate keratinocyte migration and proliferation to enhance the regeneration of the new epithelium [51]. Therefore, suppressed DF migration may induce a protracted wound-closure process, a canonical cause of keloid formation [52]. We conclude that BRCA1 repression inhibits the migration of DFs to the wound site, prolonging wound closure and further enhancing keloid formation.

NPTX2 is a PTX-encoded neuronal gene that is crucial in the formation of excitatory synapses. Its dysfunction has been implicated in various diseases of nervous system, such as Alzheimer’s disease [53] and Down’s syndrome [54]. Recent studies also widely reported associations between NPTX2 and cancer progression or treatment response, and high NPTX2 expression was closely related to enhanced cancer cell proliferation, migration, and invasion abilities [55,56]. We found that the inhibition of BRCA1 in normal DFs let to a significant decline in NPTX2 regulation. NPTX2 was also heavily regulated downwards in keloid DFs compared with normal samples. BRCA1 binds directly to an amplification region associated with the NPTX2 promoter in normal DFs but not in keloid DFs. This enhancer region showed a significantly looser organization in normal DFs than in keloid DFs, which might contribute to its repression of keloid expression. The chromatin remodeling functions of BRCA1 have rarely been reported via multiple mechanisms, such as interactions with the chromatin remodeling complex [57] or the direct influence on the structure of chromatin loop [37]. In our study, BRCA1 inhibition indeed induced the downregulation of several genes along with their loss of ATAC-seq signal in there cis-regulation elements but not most of BRCA1 targets. We speculated that certain compensatory mechanisms might contribute to the maintenance of the chromatin structure of some BRCA1 targets. Hence, we proposed that BRCA1 inhibition in normal DFs might attenuate NPTX2 enhancer accessibility, dismissing the interaction between the NPTX2 enhancer and promoter followed by NPTX2 repression, eventually resulting in slowed DF migration during the inflammation phase of wound-healing. Considering the difficulties of the current therapeutic method in eradicating keloid and the potential role of NPTX2 inhibition in keloid formation, we proposed the development of NPTX2 agonist might represent a novel option for keloid clinical treatment. However, extensive in vitro as well as in vivo experiments are indeed needed before its clinical use.

## 5. Conclusions

Chromatin represents a polymer comprising nucleic acid and protein. Chromatin structure is dynamically regulated through multiple mechanisms that underlie the transcriptional regulation. In this study, we systematically compared chromatin accessibility and transcriptional landscapes between normal and keloid DFs and uncovered the association between chromatin state transformation and gene expression dysregulation in keloid formation. Additionally, BRCA1 has been identified as a participant in regulating the structure of the chromatin polymer, and it could inhibit the wound-healing process of the wound, which in turn induces the formation of keloids by extending the wound closure process by reducing the accessibility of the NPTX2 amplifier. This will shed new light on keloid occurrence and provide clues for therapeutic development.

## Data Availability

The high-throughput sequencing dataset will be uploaded to the public database and the raw data of molecular biology experiment will be provided on request.

## Supplementary Materials

The following supporting information can be downloaded at: https://www.mdpi.com/article/10.3390/polym14163391/s1, Figure S1 Fragment length distribution of ATAC-seq of keloid (A) and normal (B) DF sample; Figure S2 Significantly enriched KEGG pathways of looser (A) and tighter (B) chromatin regions in keloid DF samples compared with that of normal samples; Figure S3 Circle plot of significantly enriched KEGG pathways (A) and GO terms (B) of DEGs in keloid DF samples compared with that of normal samples. Dots represent genes and different color represents the down- or up-regulation; Figure S4 GSEA plot of significantly enriched KEGG pathways in shBRCA1 DF samples; Figure S5 IGV visualization of ChIP-seq signal of BRCA1, H3K4me1, H3K27ac as well as ATAC-seq signal on TP63, a conventional target of BRCA1. Both the H3K4me1 and H3K27ac ChIP-seq are in keloid DF samples. H, Health; K, Keloid.
