# Supplementary material for: Comprehensive Analysis of Chromatin Accessibility and Transcriptional Landscape Identified BRCA1 Repression as a Potential Pathological Factor for Keloid
Source: Polymers (Basel). 2022 Aug 19;14(16):3391. doi: 10.3390/polym14163391 (PMC9413150; doi:10.3390/polym14163391)
Supplement: Supplementary file 1 [file polymers-14-03391-s001.zip › Supplementary Figure Legends.pdf]

Figure S1 Fragment length distribution of ATAC-seq of keloid **(A)** and normal **(B)** DF sample.

Figure S2 Significantly enriched KEGG pathways of looser **(A)** and tighter **(B)** chromatin regions in keloid DF samples compared with that of normal samples.

Figure S3 Circle plot of significantly enriched KEGG pathways **(A)** and GO terms **(B)** of DEGs in keloid DF samples compared with that of normal samples. Dots represent genes and different color represents the down- or up-regulation.

Figure S4 GSEA plot of significantly enriched KEGG pathways in shBRCA1 DF samples.

Figure S5 IGV visualization of ChIP-seq signal of BRCA1, H3K4me1, H3K27ac as well as ATAC-seq signal on TP63, a conventional target of BRCA1. Both the H3K4me1 and H3K27ac ChIP-seq are in keloid DF samples. H, Health; K, Keloid.
