# Supplementary figures and images for: Comprehensive Analysis of Chromatin Accessibility and Transcriptional Landscape Identified BRCA1 Repression as a Potential Pathological Factor for Keloid
Source: Polymers (Basel). 2022 Aug 19;14(16):3391. doi: 10.3390/polym14163391 (PMC9413150; doi:10.3390/polym14163391)

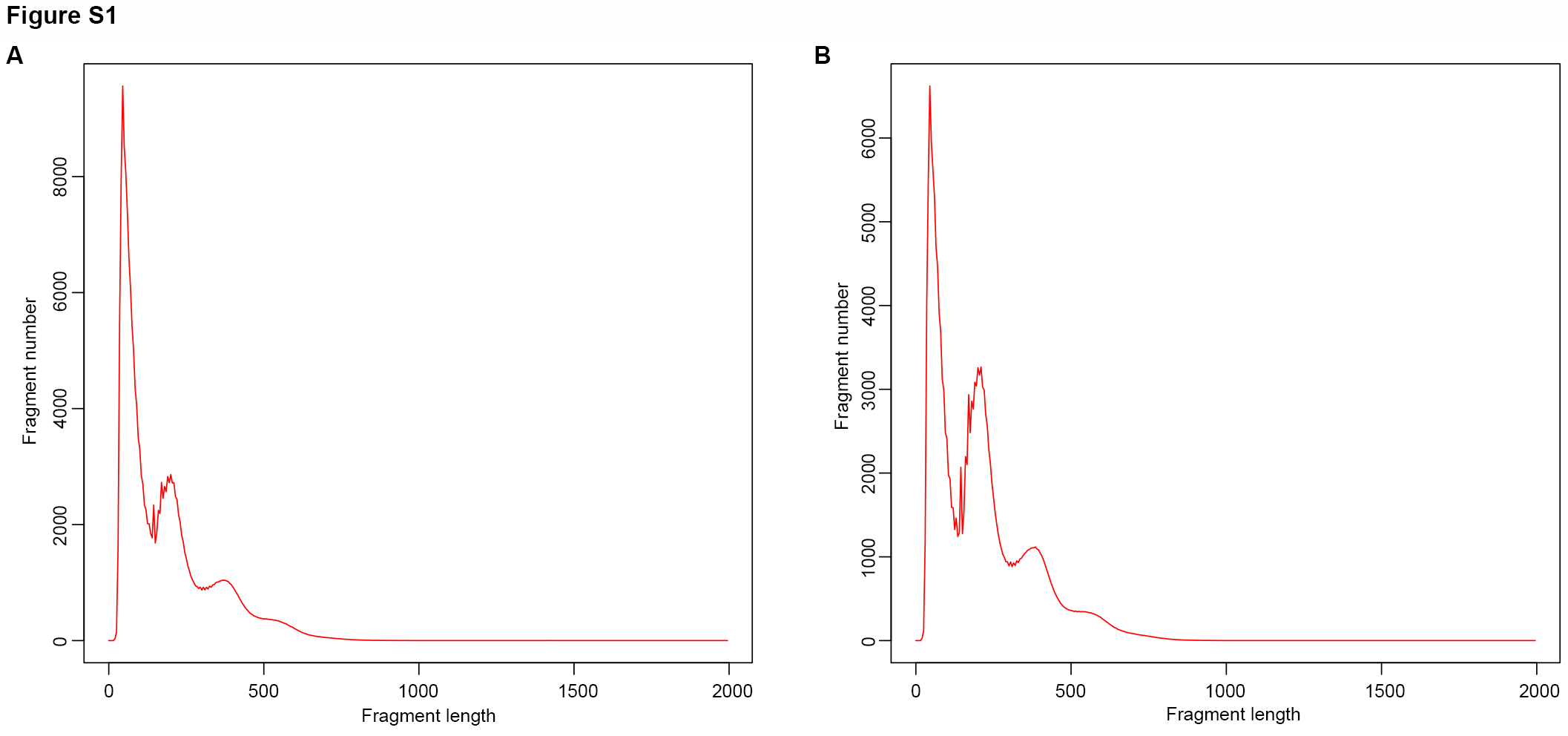

Supplement: Supplementary file 1 [file polymers-14-03391-s001.zip › Figure S1.tif]

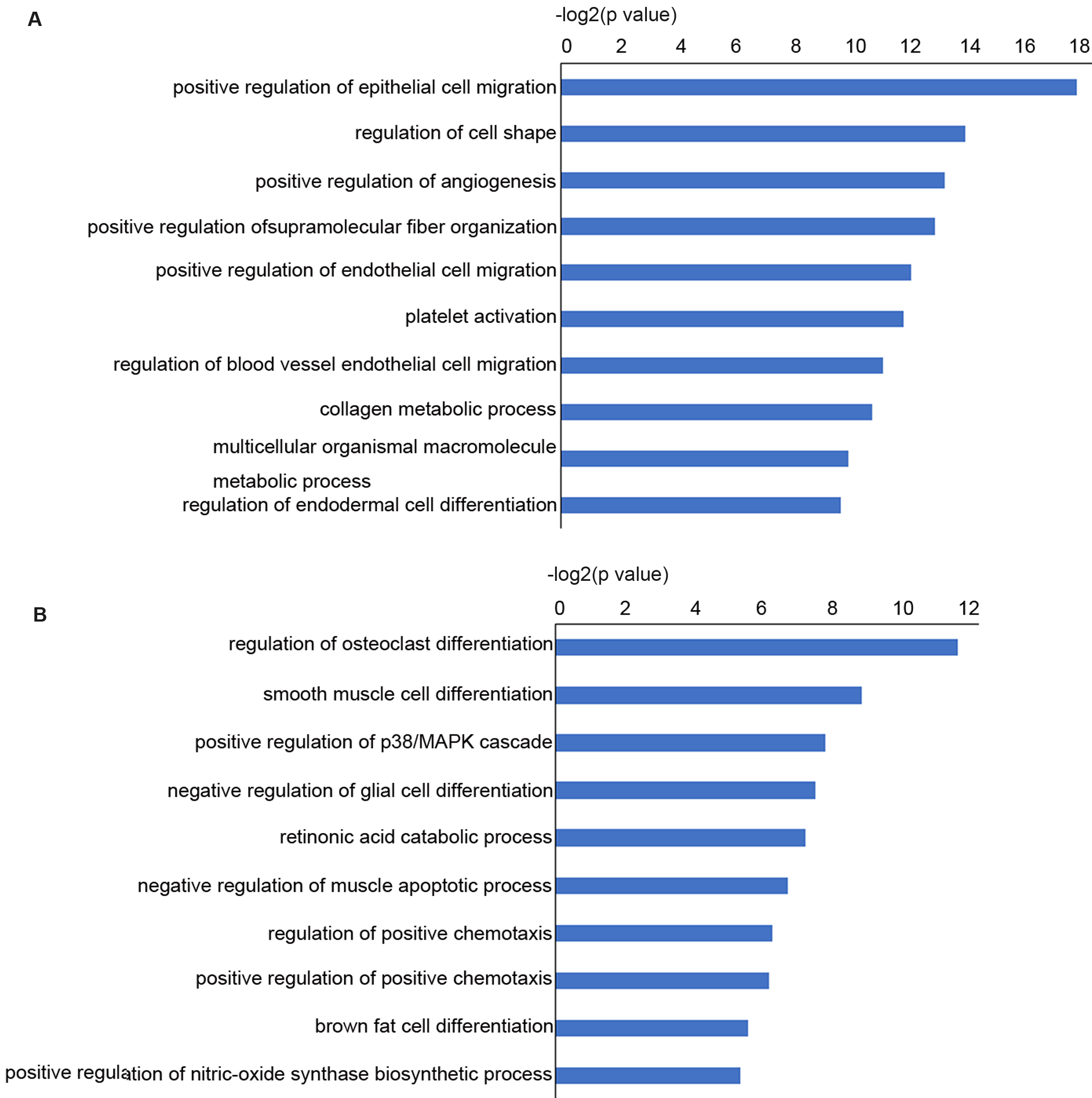

Supplement: Supplementary file 1 [file polymers-14-03391-s001.zip › Figure S2.tif]

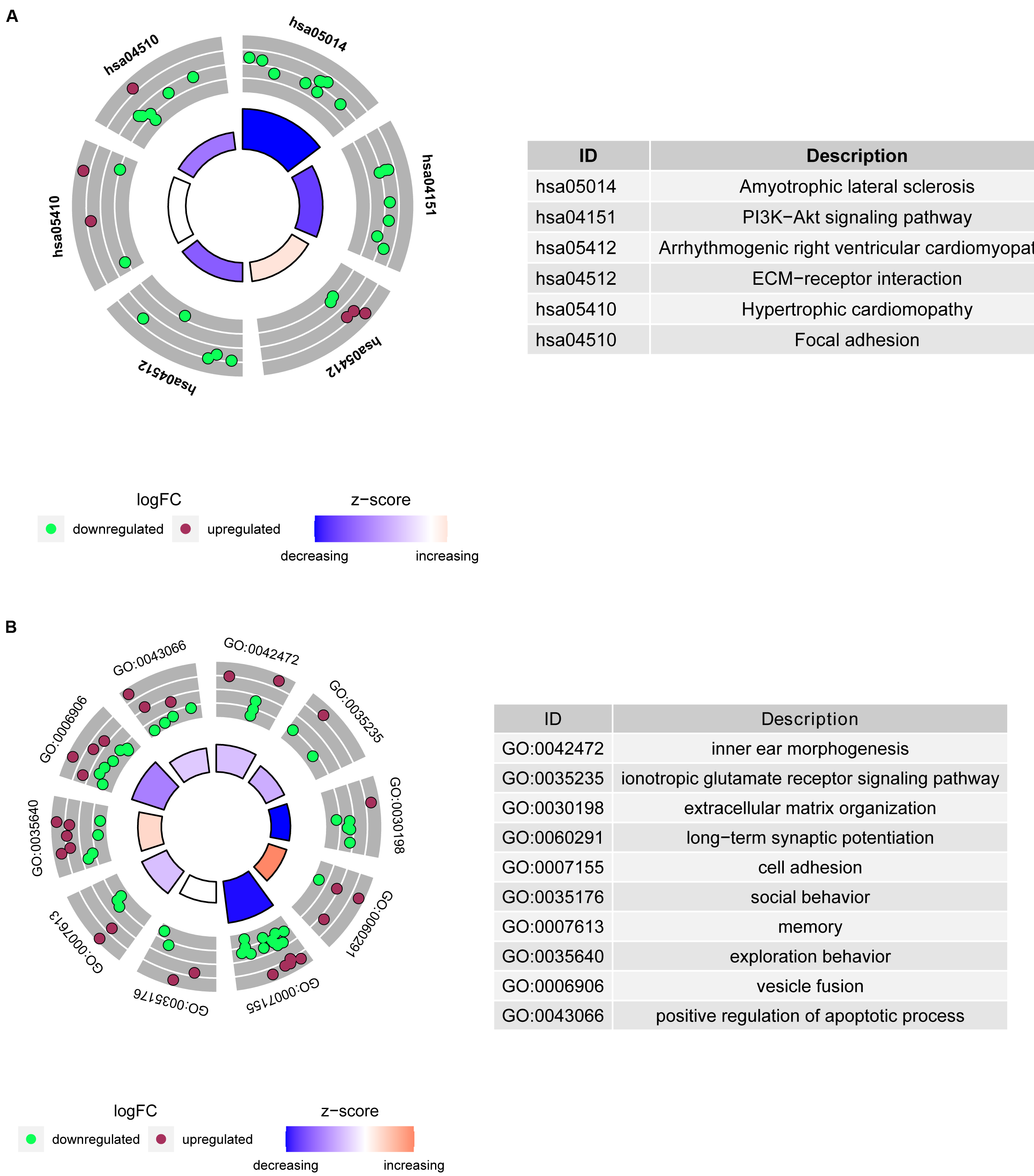

Supplement: Supplementary file 1 [file polymers-14-03391-s001.zip › Figure S3.tif]

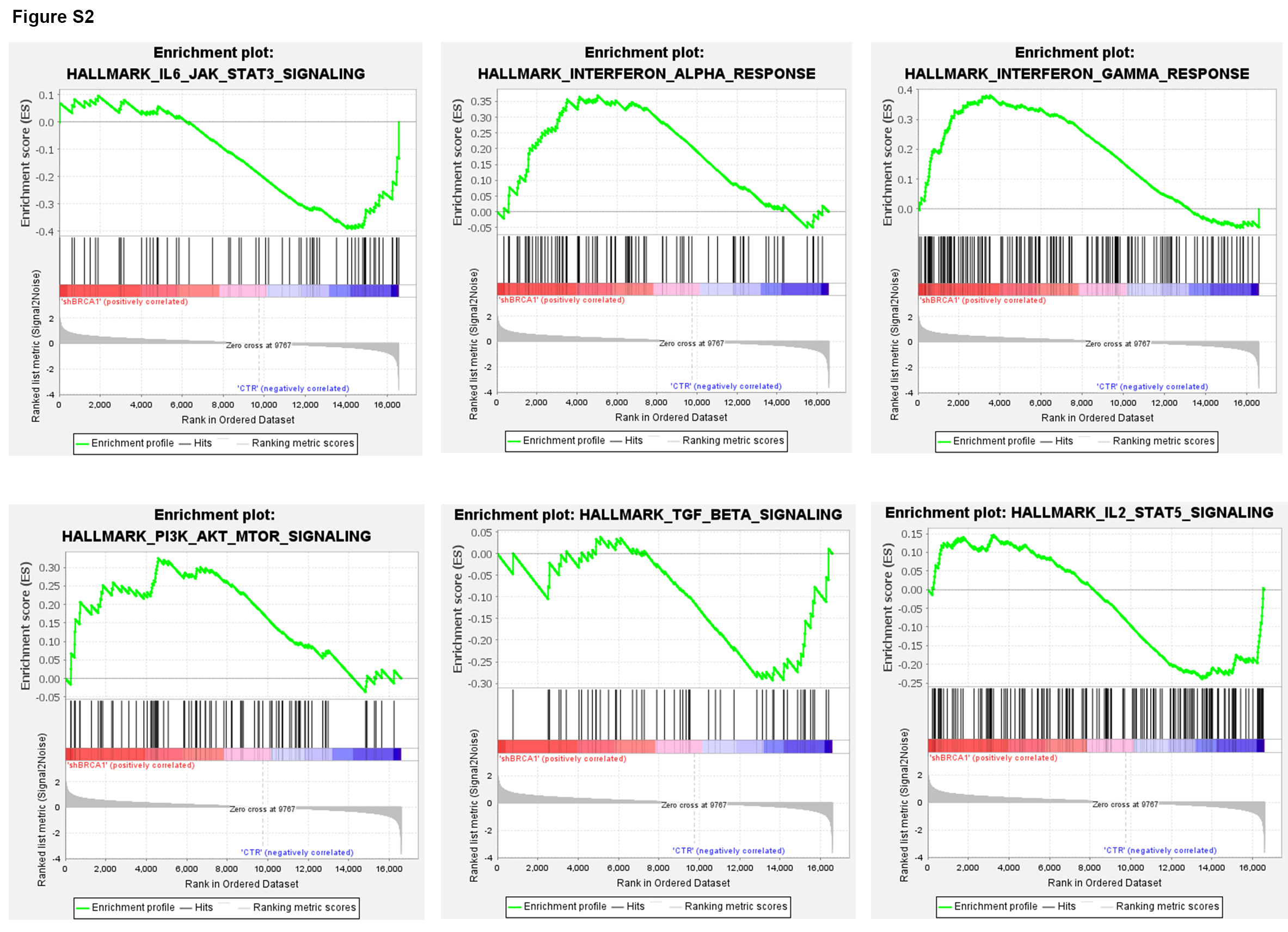

Supplement: Supplementary file 1 [file polymers-14-03391-s001.zip › Figure S4.tif]

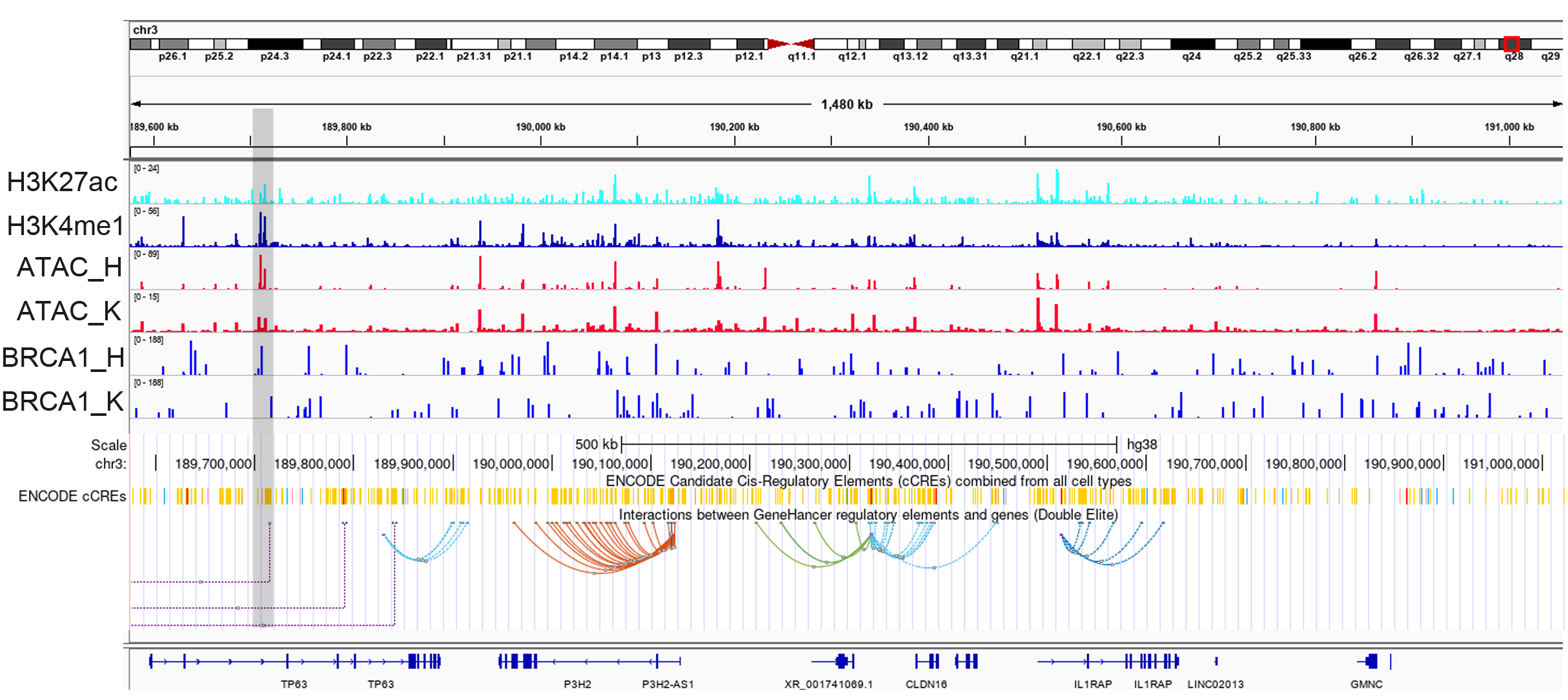

Supplement: Supplementary file 1 [file polymers-14-03391-s001.zip › Figure S5.tif]
